# Association Between Loneliness and Sleep-Related Problems Among Japanese Workers During the COVID-19 Pandemic

**DOI:** 10.3389/fpubh.2022.828650

**Published:** 2022-04-05

**Authors:** Hirofumi Tesen, Yusuke Konno, Seiichiro Tateishi, Ayako Hino, Mayumi Tsuji, Akira Ogami, Masako Nagata, Keiji Muramatsu, Reiji Yoshimura, Yoshihisa Fujino

**Affiliations:** ^1^Department of Psychiatry, University of Occupational and Environmental Health, Japan, Kitakyushu, Japan; ^2^Department of Environmental Epidemiology, Institute of Industrial Ecological Sciences, University of Occupational and Environmental Health, Japan, Kitakyushu, Japan; ^3^Department of Occupational Medicine, School of Medicine, University of Occupational and Environmental Health, Japan, Kitakyushu, Japan; ^4^Department of Mental Health, Institute of Industrial Ecological Sciences, University of Occupational and Environmental Health, Japan, Kitakyushu, Japan; ^5^Department of Environmental Health, School of Medicine, University of Occupational and Environmental Health, Japan, Kitakyushu, Japan; ^6^Department of Work Systems and Health, Institute of Industrial Ecological Sciences, University of Occupational and Environmental Health, Japan, Kitakyushu, Japan; ^7^Department of Occupational Health Practice and Management, Institute of Industrial Ecological Sciences, University of Occupational and Environmental Health, Japan, Kitakyushu, Japan; ^8^Department of Preventive Medicine and Community Health, School of Medicine, University of Occupational and Environmental Health, Japan, Kitakyushu, Japan

**Keywords:** COVID-19, loneliness, sleep, workers, Japan

## Abstract

**Background:**

The coronavirus disease 2019 (COVID-19) pandemic has been linked to a rise in loneliness. Loneliness is associated with sleep-related problems, which in turn can be a risk factor for various psychiatric disorders. However, it is unclear whether loneliness is linked to sleep-related problems during the pandemic. Here, we studied the association between loneliness and sleep-related problems during the COVID-19 pandemic in Japan.

**Methods:**

A total of 33,302 individuals who indicated they were employed were surveyed online. The survey responses of 27,036 participants were analyzed. Odds ratios (ORs) were estimated using univariate and multiple logistic regression analyses.

**Results:**

Of those analyzed, 2,750 (10.2%) experienced feelings of loneliness. Further, sleep-related problems were significantly more common among those who felt lonely both in the short term (more than 3 days) and the long term (more than 3 months). The ORs were much weaker after adjusting for factors related to interpersonal connections, such as family and friendships, than after adjusting for factors related to socioeconomic status.

**Conclusion:**

Loneliness may be a risk factor for sleep-related problems in the COVID-19 pandemic. Having connections with family and friends may have a moderating effect on the occurrence of sleep-related problems.

## Introduction

Since the first confirmed cases of coronavirus disease 2019 (COVID-19), the disease has become a major infection risk around the world. Additionally, the associated pandemic has posed numerous other public health challenges such as loneliness (1, 2). Physical distancing and curtailing outings and opportunities for socializing are some of the recommended measures for preventing infection. Specifically, governing bodies around the world have requested the public to refrain from going out as much as possible, conduct work and leisure activities at home, and refrain from socializing with those other than family members as much as possible. These recommendations are being linked to increased loneliness. One study reported that 35% of residents who experienced lockdown in China had psychological distress, while another demonstrated that 45% of adults in the US had anxiety and stress (3, 4). The circumstances of those who experience loneliness have been worsened by the pandemic (5). Further, individuals with heightened stress of anxiety and loneliness have poorer sleep quality (6).

Even before COVID-19, loneliness was an emerging public health issue. Researchers had begun to explore the possibility that loneliness may be a trigger for public health intervention for all generations (7). According to previous studies, 10–40% of the population experienced loneliness and isolation (8, 9). While isolation refers to a lack of social interaction, loneliness is linked to subjective feelings. Although different, they are related, with isolation and loneliness shown to adversely affect health through both common and different pathways (10). Loneliness is associated with lower subjective health and lower quality of life, and exacerbates signs of depression (11). It is also a risk factor for suicide and dementia (12–14).

In particularly, loneliness is associated with sleep-related problems, which in turn can be a risk factor, precursor, or accompanying symptom of various psychiatric disorders. In the COVID-19 pandemic, loneliness has been identified as a major risk factor for insomnia (15). A study in Japan on patients who visited a psychiatric clinic during the pandemic demonstrated a link between loneliness and earlier bedtime and increased sleep duration (16). Other reports suggest that sleep disorders are on the rise during the pandemic (17).

Despite reports of an increase in people experiencing loneliness and isolation during the COVID-19 pandemic, the relationship between loneliness and sleep-related problems is unclear. Here, we studied the relationship between loneliness and sleep-related problems during the COVID-19 pandemic in Japan.

## Methods

### Study Design and Participants

The present analysis forms part of the Collaborative Online Research on the Novel-Coronavirus and Work (CoroNaWork) Project, a cross-sectional study conducted between December 22 and 26, 2020, that used Internet-based surveys to probe the health of Japanese employees during the COVID-19 pandemic. A full description of the protocol is provided elsewhere (18). The survey was performed on individuals with an employment contract. Individuals whose response time was extremely short, height was below 140 cm, weight was below 30 kg, or provided conflicting answers to the same question were excluded. We excluded those with a response time of <6 min because this was considered the minimum time required to read and respond to the pre-checked text; a response time less than this was considered fraudulent. Out of 33,302 participants, responses from 27,036 were analyzed.

This study was conducted with the approval of the Ethics Committee of the University of Occupational and Environmental Health (Approval Number R2-079). Informed consent was obtained through a form on the survey website.

### Assessment of Loneliness

We used a survey item to assess participants' loneliness. The survey item asked how often the participants had felt lonely during the last 30 days. Those who answered “never” or “a little” were grouped as feeling no loneliness. In contrast, those who answered “sometimes,” “usually,” or “always” were grouped as feeling loneliness.

### Assessment of Sleep

We used a questionnaire to assess participants' sleep status. The questionnaire asked three questions. The first asked whether participants were getting enough sleep. The second asked whether they had experienced any trouble sleeping for more than 3 days. The third asked whether they had experienced any trouble sleeping for more than 3 months. Participants answered yes or no to these questions.

### Other Covariates

For analysis, we treated the following as confounding factors: age, sex, marital status, equivalent income, education smoking, alcohol consumption (demographic variables); job type, number of employees at the workplace (occupational variable); cumulative incidence rate of COVID-19 in the prefecture of residence (infection-related variable); and lack of friends to talk to, lack of acquaintances to ask for favors, lack of people to communicate with through social network sites, family time and solitary eating (social variables).

Additionally, we used the cumulative incidence of COVID-19 in the prefecture of residence in the month prior to the survey as a community-level variable. These data were taken from the websites of public institutions.

### Statistical Analysis

We identified a number of potential confounding factors in the relationship between loneliness and sleep. Multivariate analysis was used to adjust for confounding factors related to demographic background, occupational environment, and social background. Odds ratios (ORs) were estimated using univariate and multiple logistic regression analyses. Loneliness was treated as an independent variable and the presence of sleep-related problems as a dependent variable. To determine the association between loneliness and sleep problems, we constructed two multivariate models. In model 1, we adjusted for age, sex, marital status, equivalent income, education, smoking, alcohol consumption, job type, number of employees in the workplace and cumulative incidence rate of COVID-19 in the prefecture of residence. In model 2, we additionally adjusted for lack of friends to talk to, lack of acquaintances to ask for favors, lack of people to communicate with through social networking sites, family time and solitary eating.

Dummy variables were as follows: age, sex (male = 0, female = 2), marital status (married = 1, divorce/bereavement = 2, never married = 3), equivalent income (million JPY: 40–249 = 0, 250–357 = 1, 376–499 = 2, ≥500 = 3), education (junior high school = 1, high school = 2, university, graduate school, vocational school, junior college = 3), current smoke (no = 0, yes = 1), alcohol consumption (6–7 days a week = 1, 4–5 days a week = 2, 2–3 days a week = 3, <1 day a week = 4, hardly ever = 5) (demographic variables); job type (mainly desk work = 1, mainly work involving communicating with people = 2, mainly labor = 3), number of employees in the workplace (<10 = 1, <100 = 2, <1,000 = 3, >1,000 = 4) (occupational variable); cumulative incidence rate of COVID-19 in the prefecture of residence [incidence rate per million population: 97–356 = 1, 438–490 = 2, 535–911 = 3, 1,168–3,496 (non-Kanto) = 4, 1168–3,496 (Kanto) = 5] (infection-related variables); lack of friends to talk to (0 or 1), lack of acquaintances to ask for favors (0 or 1), lack of people to communicate with through social network sites (0 or 1), family time (more than 2 h = 1, more than 1 h = 2, more than 30 min = 3, <30 min = 4, almost never = 5) and solitary eating (eat alone: 6–7 days a week = 1, 4–5 days a week = 2, 2–3 days a week = 3, <1 day a week = 4, hardly ever = 5) (social variables).

All analyses were conducted using Stata (Stata Statistical Software: Release 16. College Station, TX: StataCorp LLC, United States.), with *p* <0.05 indicating statistical significance.

## Results

Table 1 summarizes the general characteristics of the 27,036 participants included in the study. Of those analyzed, 2,750 (10.2%) experienced feelings of loneliness. Age (years), mean (SD) for non-loneliness was “47.3 (10.5),” and that for loneliness was “44.5 (10.1),” respectively. Age, region, occupation, and income were comparable between those who felt lonely and those who did not. On the other hand, those who reported feeling lonely were more likely to be unmarried, divorced or bereaved.

**Table 1:** The characteristics of participants who have experienced loneliness.

**Characteristics**	**Non-loneliness**	**Loneliness**
	*n* = 24,286 (%)	*n* = 2,750 (%)
Age (years), mean (SD)	47.3 (10.5)	44.5 (10.1)
**Age group**		
20–29	1,659 (87.1%)	246 (12.9%)
30–39	4,223 (86.9%)	635 (13.1%)
40–49	7,090 (88.5%)	921 (11.5%)
50–59	8,219 (91.2%)	793 (8.8%)
60–65	3,095 (95.2%)	155 (4.8%)
Sex, male	12,601 (51.9%)	1,213 (44.1%)
**Area (cumulative COVID-19 incidence rate per million population)**		
97–356	4,767 (19.6%)	575 (20.9%)
438–490	4,903 (20.2%)	547 (19.9%)
535–911	4,765 (19.6%)	569 (20.7%)
1,168–3,496 (non-Kanto)	4,929 (20.3%)	521 (18.9%)
1,168–3,496 (Kanto)	4,922 (20.3%)	538 (19.6%)
**Marriage status**		
Married	14,077 (58.0%)	952 (34.6%)
Divorce/bereavement	2,445 (10.1%)	398 (14.5%)
Never married	7,764 (32.0%)	1,400 (50.9%)
**Job type**		
Mainly desk work	12,132 (50.0%)	1,336 (48.6%)
Mainly work involving communicating with people	6,243 (25.7%)	684 (24.9%)
Mainly labor	5,911 (24.3%)	730 (26.5%)
**Equivalent income (million JPY)**		
40–249	4,910 (20.2%)	800 (29.1%)
250–375	6,714 (27.6%)	836 (30.4%)
376–499	6,046 (24.9%)	579 (21.1%)
≥500	6,616 (27.2%)	535 (19.5%)
**Educational background**		
Junior high school	306 (1.3%)	62 (2.3%)
High school	6,190 (25.5%)	763 (27.7%)
University, graduate school, vocational school, junior college	17,790 (73.3%)	1,925 (70.0%)
Current smoke	6,274 (25.8%)	730 (26.5%)
**Alcohol consumption**		
6–7 days a week	5,179 (21.3%)	495 (18.0%)
4–5 days a week	1,910 (7.9%)	167 (6.1%)
2–3 days a week	2,935 (12.1%)	331 (12.0%)
<1 day a week	4,071 (16.8%)	476 (17.3%)
hardly ever	10,191 (42.0%)	1,281 (46.6%)
**Number of employees in the workplace**		
<10	5,619 (23.1%)	546 (19.9%)
<100	6,183 (25.5%)	757 (27.5%)
<1,000	6,379 (26.3%)	774 (28.1%)
>1,000	6,105 (25.1%)	673 (24.5%)
Do you have friends or neighbors with whom you can easily engage in small talk or daily conversation?	17,029 (70.1%)	1,057 (38.4%)
Do you have someone you can ask for help?	16,901 (69.6%)	932 (33.9%)
Do you have a partner with whom you can communicate closely using SNSs?	15,032 (61.9%)	1,136 (41.3%)
**Time spent with family having a meal or at home**		
More than 2 h	4,103 (16.9%)	272 (9.9%)
More than 1 h	5,922 (24.4%)	390 (14.2%)
More than 30 min	5,160 (21.2%)	451 (16.4%)
<30 min	3,185 (13.1%)	368 (13.4%)
Almost never	5,916 (24.4%)	1,269 (46.1%)
**How often do you eat all meals of the day alone?**		
6–7 days a week	4,276 (17.6%)	1,026 (37.3%)
4–5 days a week	2,064 (8.5%)	270 (9.8%)
2–3 days a week	2,501 (10.3%)	327 (11.9%)
<1 day a week	2,496 (10.3%)	234 (8.5%)
hardly ever	12,949 (53.3%)	893 (32.5%)
**Sleep status**		
Is your time of sleeping enough?	9,712 (40.0%)	1,766 (64.2%)
Do you have any troubles about sleep for more than 3 days?	6,469 (26.6%)	1,582 (57.5%)
Do you have any troubles about sleep for more than 3 months?	5,437 (22.4%)	1,405 (51.5%)

Table 2 summarizes the ORs of loneliness associated with sleep-related problems as estimated by the logistic model. We found a significant association between loneliness and the presence of sleep-related problems evaluated using the question “Do you get enough sleep?” The age-sex adjusted OR was 2.64 (95% CI 2.43–2.87). The association remained significant after adjusting for confounders in model 1 (OR = 2.58, 95% CI 2.37–2.80) and model 2 (OR = 2.05, 95% CI 1.89–2.24). A significant association was also observed between loneliness and the presence of short-term sleep-related problems based on the question “Have you had any trouble sleeping for more than 3 days?” The age-sex adjusted OR that participants who felt lonely had sleep-related problems was 3.63 (95% CI 3.35–3.94). The association was likewise significant in model 1 (OR = 3.53, 95% CI 3.25–3.83) and model 2 (OR = 2.95, 95% CI 2.71–3.22). Further, we also observed a significant association between loneliness and the presence of long-term sleep-related problems based on the question “Have you had any trouble sleeping for more than 3 months?” Among those who reported feeling lonely, the age-sex adjusted OR for sleep-related problems was 3.59 (95% CI 3.31–3.90). Similarly, the association was significant in model 1 (OR = 3.50, 95%CI 3.23–3.80) and model 2 (OR = 2.87, 95%CI 2.64–3.13).

**Table 2:** The association between loneliness and sleep.

	**Age-sex adjusted**				Multivariate^*^(model 1)				Multivariate^**^(model 2)			
	**OR**	**95% CI**		***p***	**OR**	**95% CI**		***p***	**OR**	**95% CI**		***p***
**slp1**
Loneliness	2.64	2.43	2.87	<0.001	2.58	2.37	2.80	<0.001	2.05	1.89	2.24	<0.001
**slp2**
Loneliness	3.63	3.35	3.94	<0.001	3.53	3.25	3.83	<0.001	2.95	2.71	3.22	<0.001
**slp3**
Loneliness	3.59	3.31	3.90	<0.001	3.50	3.23	3.80	<0.001	2.87	2.64	3.13	<0.001

* *Multivariate model further adjusted for marital status, equivalent income, educational level, smoking, alcohol consumption, job type, number of employee at the workplace and cumulative incidence rate of COVID-19 at prefecture.*

** *Multivariate model further adjusted for lack of friends to talk to, lack of acquaintances to ask for favors, lack of people to communicate with through social networking sites, family time and solitary eating.*

*slp1: Is your time of sleeping enough?*

*slp2: Do you have any troubles about sleep for more than 3 days?*

*slp3: Do you have any troubles about sleep for more than 3 months?*

## Discussion

We found that, during the COVID-19 pandemic, sleep-related problems were significantly more common among those who felt lonely both in the short term (more than 3 days) and the long term (more than 3 months). The OR of loneliness associated with sleep-related problems was much weaker when adjusted for factors related to interpersonal connections, such as family and friendships, than when adjusted for factors related to socioeconomic status. This suggests that having connections with family and friends has a moderating effect on the occurrence of sleep-related problems.

About 10% of participants in this study felt lonely. To our knowledge, this is the first large-scale study to investigate loneliness in a working population in Japan. A previous study based on 15,530 ordinary people in the UK also reported an association of similar risk factors with loneliness and mental illness (19). Our study is significant in that it examined an even larger number of employees (*n* = 27,036) in Japan. Further, in contrast to the finding that having a job is a protective factor against loneliness and mental illness in the UK study, we found that a marked number of people in Japan felt lonely despite having a job. We also examined additional risk factors. According to a previous Japanese study, the percentage of individuals experiencing loneliness among those aged 65 and above who were living with a spouse only, living with children, and living alone was 17.7, 18.5, and 37.3%, respectively (20). The lower incidence of loneliness in the present study may reflect the fact that working-age individuals more actively participate in society through work, and are in the early stages of marriage and raising children. However, we found that workers who were unmarried, divorced, or had lost a partner; had no neighbors or friends to talk, ask for favors, or communicate with on social networking sites; had little time to spend with family, or ate meals alone tended to feel lonely despite working.

Our analyses showed that those who felt lonely typically had sleep-related problems. These results are consistent with those of previous studies. One report found that pandemic-related loneliness, anxiety, and depression led to insomnia, which is more pronounced among women and inner-city residents. The study examined the association between loneliness and insomnia in 2,427 ordinary people in Greece (17). Our study is novel in its large-scale nature, investigating loneliness and sleep-related problems in 27,036 workers in Japan, who are considered to be socially engaged on a regular basis. A report on 556 members of the general public in France also showed that pandemic-related loneliness and anxiety were associated with insomnia (15), with 19.1% reporting insomnia. This figure is half that reported in Greece, but comparable to that reported in China and Italy (21, 22). In our study, we found that 10.2% of Japanese workers felt lonely. Loneliness has been shown to be associated with sleep fragmentation and poor sleep quality (23). A study that adjusted for the effects of depressive symptoms suggested that the relationship between loneliness and insomnia cannot be explained by the comorbidity of depressive symptoms alone (24). When individuals experience loneliness and threats to the safety of the social environment, vigilance against social threats is enhanced and the brain remains alert during sleep (25). Those who maintain good relationships with others tend to choose healthy behavioral actions (26). Having social relationships and choosing healthy behaviors has been suggested to lead to good sleep quality (27).

Our study investigated the relationship between loneliness and sleep-related problems in the context of the COVID-19 pandemic, where people are being asked to refrain from unnecessary movement and physical interaction. A study of 34,484 workers in the UK reported that a flexible schedule and telework improve work-family balance, increase job satisfaction, especially among women, and have mental health benefits (28). However, it is also possible there is evidence that telework may be associated with loneliness, and as a consequence, sleep-related problems. This needs further study.

We found that having interpersonal connections with family and friends was effective in alleviating sleep-related problems in workers who felt lonely during the COVID-19 pandemic. The significant association of loneliness with sleep problems was true even after accounting for socioeconomic factors such as sex, age, and marriage. However, further adjusting for interpersonal connections with family and friends in model 2 led to a marked attenuation of the OR of sleep-related problems, indicating that the relationship between loneliness and sleep-related problems can be partially explained by the adjusted factors. To prevent spread of COVID-19 in Japan, the government has requested that people engage in physical distancing and refrain from going out. Self-isolation has been encouraged, for example, by performing work and leisure activities at home and refraining from interacting with those outside the family as much as possible. These requests may have brought the problem of loneliness to the surface for some workers. For those who live with their families, self-isolation allows them to spend more time and strengthen relationships with their kin. However, for workers who live alone or have no community ties outside of work, self-isolation may enhance the negative effects of loneliness.

Our study has several limitations. First, because this study was conducted on Internet users, the degree to which the results are generalizable is unclear. To reduce bias, we sampled based on region, job type and prefecture according to the rate of infection. We also considered the common-method variance bias, because internet surveys frequently use standardized question options. However, we judged that any common-method variance bias would be small because the Harman's one-factor test on all self-reported outcome measures used, namely, the Kessler 6 scale, Work Functioning Impairment Scale, and Job Content Questionnaire, explained 25% of the variance, which is lower than 50%. We also tried to reduce desirability bias by blinding the researchers to the results to ensure anonymity and confidentiality. The survey was also computer-controlled. Further, desirability bias is generally a problem in reports of ability, personality, sexual behavior, and drug use (29), and thus was unlikely to be a significant issue in our study. Meanwhile, recall bias is especially problematic in retrospective studies that aim to explore the etiology of mental states. There may have been recall bias in our study because it examined varying degrees of loneliness and sleep-related problems. As we were unable to determine causality, it is possible that those with sleep-related problems complain of loneliness. Second, whether or not participants felt lonely was determined using one question: “During the last 30 days, how often did you feel the following emotions?” There are variety ways to evaluate loneliness; in this study, we assessed loneliness by asking participants how often they felt lonely in the past 30 days. This method was adapted from a previous study that assessed loneliness using a single question (30). We feel that the question is appropriate as it briefly asks about participants' subjective experience. Further studies using less subjective assessments of loneliness are needed to confirm our findings. Third, we were unable to assess the severity of sleep problems as we did not use the insomnia rating scale. We used three questions to assess sleep problems, the reliability and validity of which are uncertain. However, the three questions inquired about participants' symptoms over 3 days and 3 months based on the Diagnostic and Statistical Manual of Mental Disorders, 5th edition (DSM-5), for insomnia, and the alpha coefficient was relatively high at 0.78. In addition to DSM-5, the three questions were also developed with reference to the Athens Insomnia Scale, both of which are widely used around the world. We made the questions simple but appropriate for understanding sleep-related problems. Finally, because this was a cross-sectional study, we could not determine the temporal or causal link between loneliness and sleep-related problems; the results are purely correlational.

In conclusion, loneliness was found to be a risk factor for sleep-related problems during the COVID-19 pandemic. Our findings suggest that having connections with family and friends has a moderating effect on the occurrence of sleep-related problems. However, it is not yet clear whether family and friendship-related interventions will be effective. Further studies are needed to provide causal evidence for the relationship and confirm the effectiveness of such interventions. Further, as workers who have no connections with family and friends are at high risk of sleep problems, identifying workers who feel lonely and have reduced opportunities for direct communication during the pandemic may prevent adverse downstream effects. Given the pandemic is still ongoing, strategies are needed to manage loneliness and sleep-related problems.

## Data Availability Statement

The original contributions presented in the study are included in the article/supplementary material, further inquiries can be directed to the corresponding author.

## Ethics Statement

The studies involving human participants were reviewed and approved by the Ethics Committee of the University of Occupational and Environmental Health (Approval Number R2-079). The patients/participants provided their written informed consent to participate in this study.

## Author Contributions

YF was the chairperson of the study group. YK conceived the research questions. HT conducted the statistical analysis with YF and YK. HT drafted the initial manuscript. All the authors designed the research protocol, developed the questionnaire, revised, and approved the final manuscript.

## Funding

This study was supported by a research fund from the University of Occupational and Environmental Health, Japan; Japanese Ministry of Health, Labor and Welfare (H30-josei-ippan-002, H30-roudou-ippan-007, 19JA1004, 20JA1006, 210301-1, and 20HB1004); General Incorporated Foundation (Anshin Zaidan), the Collabo-Health Study Group and Hitachi Systems, Ltd., and scholarship donations from Chugai Pharmaceutical Co., Ltd. The funder was not involved in the study design, collection, analysis, interpretation of data, the writing of this article, or the decision to submit it for publication.

## Conflict of Interest

The authors declare that the research was conducted in the absence of any commercial or financial relationships that could be construed as a potential conflict of interest.

## Publisher's Note

All claims expressed in this article are solely those of the authors and do not necessarily represent those of their affiliated organizations, or those of the publisher, the editors and the reviewers. Any product that may be evaluated in this article, or claim that may be made by its manufacturer, is not guaranteed or endorsed by the publisher.
